# Book Review: Evolving Enactivism: Basic Minds Meet Content

**DOI:** 10.3389/fpsyg.2017.01947

**Published:** 2017-11-03

**Authors:** Ed Baggs

**Affiliations:** ^1^Bartlett School of Architecture, University College London, London, United Kingdom; ^2^Department of Psychology, University of Cincinnati, Cincinnati, OH, United States

**Keywords:** antirepresentationalism, ecological psychology, dynamical systems theory, enactivism, relationalism, cognitivism, radical embodied cognitive science

Railing against cognitivism is a noble pastime. Maybe even a heroic one. Some of us believe that cognitivism hasn't achieved all that much given its hegemonic status these last decades within the sciences of the mind. Some of us wouldn't be sorry to see the back of it.

What we need, though, is a positive project with which to replace cognitivism. Something that coalesces around a core set of ideas that almost everyone agrees on as necessary starting assumptions. You know, like the kind of thing people used to call a “paradigm.”

This is ostensibly what Daniel Hutto and Erik Myin set out to achieve in their new book. Following the ground-clearing work of their earlier volume (Hutto and Myin, 2013), they now seek to present “REC's positive vision of cognition,” and to demonstrate how “REC puts its positive story into action.” (REC still stands for Radical Enactive, Embodied account of Cognition.)

H&M acknowledge that cognitivism is a formidable opponent precisely because it already has the kind of core set of ideas we're after: it is committed to representationalism and computationalism about mind, and to the view that explanations of mental phenomena should be mechanistic (i.e., that explanations should identify discrete component parts and the sequence of causal connections between them; contrast this with dynamical explanations which do not have this linear structure).

What does REC have to offer in place of these? Essentially, H&M offer a developmental story. Their claim is that non-basic, content-involving minds—that is, minds that are capable of using language, reasoning about absent entities, etc.—develop from basic minds through the mastery of “socio-cultural practices” in childhood. H&M call this their “duplex account” of cognition: a “nonbasic mind” is built on top of the basic, like a second storey built on top of a bungalow. Theirs is a “multi-storey story.” (It is an appealing story, and it is compatible with a strand of early twentieth-century theorizing, notably by Lev Vygotsky and G. H. Mead. Neither of these figures is mentioned by H&M.)

Having a story is good. But what comes next? How are we to take this story and turn it into a productive programme of research? H&M align themselves with three existing empirical programmes: autopoietic adaptive enactivism, ecological psychology, and dynamical systems theory. These approaches are united, for H&M, less by what they share than by what they reject. Each approach is compatible with antirepresentationalism about basic minds, or with the view that “enactive, embodied activity does not always and everywhere involves [sic] thinking about the world in contentful ways.” But if we wish to build a post-revolutionary cognitive science, is it enough to say that we are *against* representations?

What's most frustrating about reading H&M is that the things they criticize their opponents for are the very things they themselves are frequently guilty of. They sneer at “the general tendency of philosophers—especially those in some wings of the analytic tradition—to assume that the essence of phenomena can be investigated independently of science.” Yet when H&M themselves appeal to empirical findings it can feel as if they are doing so only for rhetorical effect. Little effort is made to explain the research to the reader: What was investigated? How was it done? The authors typically rely on second-hand descriptions of research findings from the writings of other philosophers, or at best they'll rely on the original researchers' descriptive summary.

Worse, when the authors try to identify empirical work that exemplifies the REC way of doing science they draw a blank, and feel compelled to tell us that to buy into the REC programme at this point “is still to place one's bets on an emerging rather than established paradigm.” This is an odd admission. Are examples of good REC-type science really so hard to find? All three of the approaches the authors align themselves with—dynamical, ecological, autopoietic—have been producing empirical findings for over 30 years. Why not tell us about some of this work? (See, e.g., Schöner, 2008; Chemero, 2009; Di Paolo et al., 2017).

Three chapters toward the end of the book seek to co-opt existing strands of research from mainstream cognitive science and to bring them into REC's remit. Thus H&M argue that REC is compatible with currently popular accounts of perceiving, imagining, and remembering. A chapter is given over to each. The conclusion is always the same. Respectively: predictive coding accounts of perceiving, mental modeling accounts of imagining, and social interaction theory accounts of remembering—all are compatible with antirepresentationalism about basic minds.

The question that H&M seek to address is an important one, even if their answers are underwhelming. The question bears repeating: what *should* be at the core of a non-cognitivist approach to the mind? Antirepresentationalism is not enough here. The problem with antirepresentationalism is that it does not tell us how to ask new empirical questions, only how not to ask them. An attractive alternative response is this: relationalism. What the three embodied approaches do have in common is a concern with relational structure within a cognitive system. Specifically these approaches target the animal–environment relation, or the fit between an organism and its surroundings. This is the shift in thinking that is required to get beyond cognitivism. Traditional cognitive science asks questions of the form, What representations are implicated in the production of this behavior? Relationalists instead ask, What structure in this animal–environment system is being used in the regulation of this activity? There is important philosophical work to be done in clarifying how best to go about asking this second type of question. H&M do not address this problem.

H&M's book is so doggedly philosophical-in-the-analytic-sense that it is hard to imagine how it could be translated into a positive empirical programme. The science, though, will continue to make progress. H&M should engage with it.

## Author contributions

The author confirms being the sole contributor of this work and approved it for publication.

### Conflict of interest statement

The author declares that the research was conducted in the absence of any commercial or financial relationships that could be construed as a potential conflict of interest.

## References

[B1] Chemero A. (2009). Radical Embodied Cognitive Science. Cambridge, MA: MIT Press.

[B2] Di Paolo E. A. Buhrmann T. Barandiaran X. E. (2017). Sensorimotor Life: an Enactive Proposal. Oxford: Oxford University Press.

[B3] Hutto D. D. Myin E. (2013). Radicalizing Enactivism: Basic Minds without Content. Cambridge, MA: MIT Press.

[B4] Schöner G. (2008). Dynamical systems approaches to cognition, in The Cambridge Handbook of Computational Psychology, ed Sun R. (Cambridge: Cambridge University Press), 101–126.

